# Disposal of Unused Postoperative Opioids: A Real-World Demonstration of Surgeon-initiated Strategies Using an Activated Charcoal Bag System

**DOI:** 10.1097/AS9.0000000000000313

**Published:** 2023-08-17

**Authors:** Madhuri V. Nishtala, Aos S. Karim, David Melnick, Therese Delarwelle, Elise Lawson, Caprice C. Greenberg, Tudor Borza

**Affiliations:** From the * Department of Surgery), University of Wisconsin, Madison, WI; † Door County Medical Center, Sturgeon Bay, Wisconsin; ‡ Wisconsin Surgical Outcomes Research Program (WISOR), University of Wisconsin, Madison, WI; § Department of General Surgery, University of North Carolina, Chapel Hill, NC; ‖ Department of Urology, University of Wisconsin, Madison, WI.

**Keywords:** opioid stewardship, opioid disposal, surgery

## Abstract

Excessive opioid prescribing following surgery creates a reservoir of unused medications available for diversion and abuse. We conducted a cohort study examining the impact of clinic-based, surgeon-initiated strategies using an activated charcoal bag (ACB) system on disposal of unused opioids. Among patients undergoing a variety of general surgery procedures, 67% of those with unused opioids disposed of them using the ACB. Our findings demonstrate practical ways to incorporate opioid disposal into surgical practice as a complement to judicious opioid prescribing.

The national opioid epidemic continues to worsen, leading to over 80,000 deaths in 2021 and costing the US $1.5 trillion in 2020 alone.^1,2^ Despite extensive efforts to address this, deaths from prescription opioids have not decreased since the start of the epidemic.^1^ Excess opioid prescribing remains the primary contributor to prescription opioid abuse, as this practice creates a reservoir of unused opioids available for community diversion and illicit use.^3^ Although improving, excess prescribing following surgery remains prevalent, making this a significant component of the current opioid epidemic.^4,5^ Much of the attention to date has rightly focused on decreasing excess surgical prescribing, but increasing safe disposal of unused opioids offers another path to reducing the supply of opioids available for diversion.

Current opioid disposal strategies, like drug-take back programs and “flush” lists, are perceived as inconvenient, limited by poor access and environmental concerns.^6^ Use of a drug disposal system consisting of a biodegradable activated charcoal bag (ACB)^7^ distributed perioperatively was associated with a nearly four-fold increase in self-reported disposal of unused opioids.^8^ Although effective, this approach requires significant coordination at the hospital level, which is a barrier to widespread community adoption. We examined the impact of clinic-based, surgeon-initiated strategies using the ACB system in the preoperative and postdischarge settings on disposal of unused opioids.

## METHODS

Patients were recruited at three surgical clinics: Door County Medical Center (DCMC), a community general surgery practice; University of Wisconsin (UW) Breast Center, an academic breast cancer clinic; and the UW 1SP General Surgery, an academic-affiliated clinic with regional catchment. In an effort to maximize generalizability of the findings, consecutive patients presenting for a qualifying appointment at each individual clinic were enrolled. Qualifying appointments were either postoperative or preoperative, based on the site-specific disposal strategy. Two disposal strategies were implemented. DCMC patients were instructed to bring unused opioids to their postoperative appointment, offered the ACB (Deterra; Verde Environmental Technologies, Minneapolis, MN; cost $4.28 per disposal bag)^7^ and were encouraged to dispose of unused opioids in clinic. UW patients received the ACB and an informational pamphlet regarding opioid safety at their preoperative appointment. Postoperative opioid prescription information was collected through the Electronic Health Record. Opioid dose prescribed was converted to morphine milligram equivalents (MME) to allow comparison across prescription types.^9^ Patient reported opioid use and disposal was collected at the postoperative appointment or by phone 4–6 weeks postoperatively. This study was IRB exempt under the category of quality improvement by the University of Wisconsin.

## RESULTS

One hundred nineteen patients undergoing elective surgical procedures (46 DCMC, 73 UW) were enrolled in the study between January and August 2020. Patient acceptability of the study was high, measured by both patient comments in the phone interviews and from the low number of patients declining to participate. No patients declining to participate at the DCMC or UW 1SP General Surgery clinics, and only 3 patients at the UW Breast Center clinic declining, with all citing sensitivity of their cancer diagnosis as the reason. A total of 116 patients were prescribed 1,146 opioid tablets (7,053 MME, Table 1). The median prescription size was 10 tablets at DCMC and 9 tablets at the UW sites. All DCMC patients received prescriptions and 41 (89%) had unused opioids totaling 302 tablets (1,715 MME). At UW sites, 70 patients received prescriptions with 34 (47%) having unused opioids totaling 177 tablets (1,072 MME).

**TABLE 1.** Patient, Prescription and Disposal Characteristics by Site

	Door County Medical Center (n = 46)	UW Breast Center (n = 34)	UW General Surgery (n = 39)
**Patient and procedure characteristics**			
Age (median, years)	63	56	55
Female Sex (%)	24 (52%)	33 (97%)	22 (56%)
Procedure (n, %)			
Laparoscopic appendectomy	11 (24%)	0 (0%)	2 (5%)
Laparoscopic cholecystectomy	9 (20%)	0 (0%)	12 (31%)
Lumpectomy	4 (9%)	22 (65%)	5 (13%)
Mastectomy	0 (0%)	9 (26%)	1 (3%)
Colectomy	3 (6%)	0 (0%)	1 (3%)
Inguinal hernia repair	8 (17%)	0 (0%)	7 (18%)
Other	11 (24%)	3 (9%)	11 (28%)
**Opioid prescribing characteristics**			
Patients receiving opioid prescription (%)	46 (100%)	32 (94%)	38 (97%)
Median tablets prescribed (n)	10	9	9
Patients with unused opioids (n,%)	41 (89%)	11 (32%)	23 (59%)
***Median tablets unused(n***)	6	5	6
Total number of prescribed tablets (MME)	533 (3,029)	284 (2,072)	329 (1,952)
Total number of unused tablets (MME)	302 (1,715)	66 (482)	111 (590)
**Opioid disposal characteristics among patients with unused medication**			
Patients disposing of unused opioids (n,%)	27 (66%)	8 (73%)	17 (74%)
Disposal using activated charcoal bag system (n,%)	27 (66%)	7 (64%)	16 (70%)
***Median number of tablets disposed (n***)	7	5	6
Total opioids disposed with bag (MME)	207 (1,139)	39 (292)	60 (370)
Total opioids disposed (all methods) (MME)	207 (1,139)	49 (367)	66 (400)

Italics are referring to sub-categories under the opioid prescribing characteristics and disposal characteristics categories. There is no further significance.

Overall, 52 of 75 (69%) patients with unused opioids disposed of them, 50 of these using the ACB. Disposal rates were similar between sites (66% DCMC, 74% UW) resulting in the destruction of 322/479 tablets (67%, 1,906 MME). Patients rated the experience of using the ACB as “very easy” to “easy” and 91% described their sentiment as “very positive” or “positive.” Clinics rated the disposal system as highly feasible with negligible disruption to workflow. Due to COVID-19 restrictions, postoperative appointments at DCMC transitioned to virtual visits in March, limiting patients from receiving the planned intervention.

## DISCUSSION

This study demonstrates the feasibility of clinic-based, surgeon-initiated distribution of an activated charcoal disposal bag system for safe disposal of unused opioids using two techniques. Both led to significant disposal were well received by patients and clinical staff and integrated well with clinical workflow. Patients with unused opioids overwhelmingly chose the activated charcoal bag system over other disposal methods.

Our disposal rates were well above those previously reported in the absence of an intervention and comparable to the prior randomized trial.^8^ These results further validate the use of ACB as an effective means for disposing unused opioids following surgery. Addition of the ACB is an excellent complement to judicious opioid prescribing practices in the prevention of diversion for nonmedical use.

The novel approaches initiating distribution of ACB in the surgical clinic as opposed to the hospital setting facilitate broader community dissemination. These approaches require minimal resources to implement and rely on fewer staff and coordination compared with hospital-based interventions. The cost of this intervention is low, arising primarily from the price of the ACB, which is inexpensive, and the brief added counseling time by the nurse or medical assistant. They are particularly advantageous in settings where surgeons operate at multiple sites. Further, these approaches directly leverage the trust of the patient-surgeon relationship, an important factor contributing to the high disposal rates observed. Finally, this study was conducted at the height of COVID-19 restrictions in our state, further demonstrating the feasibility of our approaches and the desire by both patients and surgeons for simplified approaches to disposal of unused opioids.
